# HIV disease, metabolic dysfunction and atherosclerosis: A three year prospective study

**DOI:** 10.1371/journal.pone.0215620

**Published:** 2019-04-18

**Authors:** Hann Low, Anh Hoang, Tatiana Pushkarsky, Larisa Dubrovsky, Elizabeth Dewar, Maria-Silvana Di Yacovo, Nigora Mukhamedova, Lesley Cheng, Catherine Downs, Gary Simon, Maria Saumoy, Andrew F. Hill, Michael L. Fitzgerald, Paul Nestel, Anthony Dart, Jennifer Hoy, Michael Bukrinsky, Dmitri Sviridov

**Affiliations:** 1 Baker Heart and Diabetes Institute, Melbourne, VIC, Australia; 2 Department of Microbiology, Immunology and Tropical Diseases, George Washington University, Washington, DC, United States of America; 3 The Heart Centre, Alfred Hospital, Melbourne, VIC, Australia; 4 HIV and STD Unit, Infectious Disease Service, Hospital Universitari de Bellvitge, Instituto de Investigación Biomédica de Bellvitge, Hospitalet de Llobregat, Barcelona, Spain; 5 Department of Biochemistry and Genetics, La Trobe Institute for Molecular Science, La Trobe University, Bundoora, VIC, Australia; 6 Department of Infectious Diseases, Alfred Hospital, Melbourne, VIC, Australia; 7 Division of Infectious Diseases, Department of Medicine, George Washington University, Washington, DC, United States of America; 8 Lipid Metabolism Unit, Centre for Computational and Integrative Biology, Massachusetts General Hospital, Harvard Medical School, Boston, MA, United States of America; 9 Department of Medicine, Monash University, Melbourne, VIC, Australia; Universiteit van Amsterdam, NETHERLANDS

## Abstract

HIV infection is known to be associated with cardiometabolic abnormalities; here we investigated the progression and causes of these abnormalities. Three groups of participants were recruited: HIV-negative subjects and two groups of treatment-naïve HIV-positive subjects, one group initiating antiretroviral treatment, the other remaining untreated. Intima-media thickness (cIMT) increased in HIV-positive untreated group compared to HIV-negative group, but treatment mitigated the difference. We found no increase in diabetes-related metabolic markers or in the level of inflammation in any of the groups. Total cholesterol, low density lipoprotein cholesterol and apoB levels were lower in HIV-positive groups, while triglyceride and Lp(a) levels did not differ between the groups. We found a statistically significant negative association between viral load and plasma levels of total cholesterol, LDL cholesterol, HDL cholesterol, apoA-I and apoB. HIV-positive patients had hypoalphalipoproteinemia at baseline, and we found a redistribution of sub-populations of high density lipoprotein (HDL) particles with increased proportion of smaller HDL in HIV-positive untreated patients, which may result from increased levels of plasma cholesteryl ester transfer protein in this group. HDL functionality declined in the HIV-negative and HIV-positive untreated groups, but not in HIV-positive treated group. We also found differences between HIV-positive and negative groups in plasma abundance of several microRNAs involved in lipid metabolism. Our data support a hypothesis that cardiometabolic abnormalities in HIV infection are caused by HIV and that antiretroviral treatment itself does not influence key cardiometabolic parameters, but mitigates those affected by HIV.

## Introduction

HIV infection is accompanied by a range of metabolic co-morbidities, with cardiovascular complications being among the most important in terms of morbidity and mortality [1–5]. Initially, cardiovascular co-morbidities of HIV infection were attributed to dyslipidaemia caused by protease inhibitor-containing treatment regimens [6, 7]. However, while the pro-atherogenic regimens were gradually phased out as initial therapy, the cardiovascular and metabolic complications persisted [8, 9]. We have suggested that HIV infection itself can cause atherosclerosis and proposed a mechanism whereby the HIV protein Nef reduces the efficiency of reverse cholesterol transport causing accumulation of cholesterol in macrophages and hypoalphalipoproteinemia [10, 11]. We also demonstrated that extracellular Nef, mimicking Nef released from HIV infected cells into circulation, caused atherosclerosis and dyslipidaemia *in vivo* in the absence of the infection or any other HIV-related factors [12]. While experimental findings point to the important role of HIV itself in the pathogenesis of cardiovascular and metabolic co-morbidities of HIV infection, separation between the effects of HIV and of antiretroviral treatment in the clinical setting is difficult as the overwhelming majority of HIV-positive patients now commence antiretroviral treatment immediately after diagnosis. Furthermore, HIV-positive patients often have overrepresented conventional cardiovascular risk factors, such as high rates of smoking, which may also contribute to the elevated cardiovascular risk [13]. These factors complicate investigation of the causes, pathogenic mechanisms and progression of HIV-associated cardiometabolic co-morbidities.

Most clinical studies investigating the development of cardiovascular disease in the context of HIV infection were cross-sectional [2, 14–17], retrospective [18], or short-term prospective studies [19–22]; they rarely included patients not treated with antiretroviral regimens. SMART, the only large, outcome based prospective study, while confirming the higher rate of cardiovascular co-morbidities in HIV patients, did not conclusively resolve the issue of its causes [23]. We recently published a prospective study comparing development of atherosclerosis in treated and untreated patients with HIV [21]. However, that study reported only 1 year of follow up and did not include an HIV-negative group. Here, we report the outcomes of a new study, which compared development of atherosclerosis, as well as changes in lipid and glucose metabolism, in HIV-negative and HIV-positive subjects with or without antiretroviral treatment over 3 years of follow up.

## Patients and methods

### Participant recruitment

**Fig 1:**
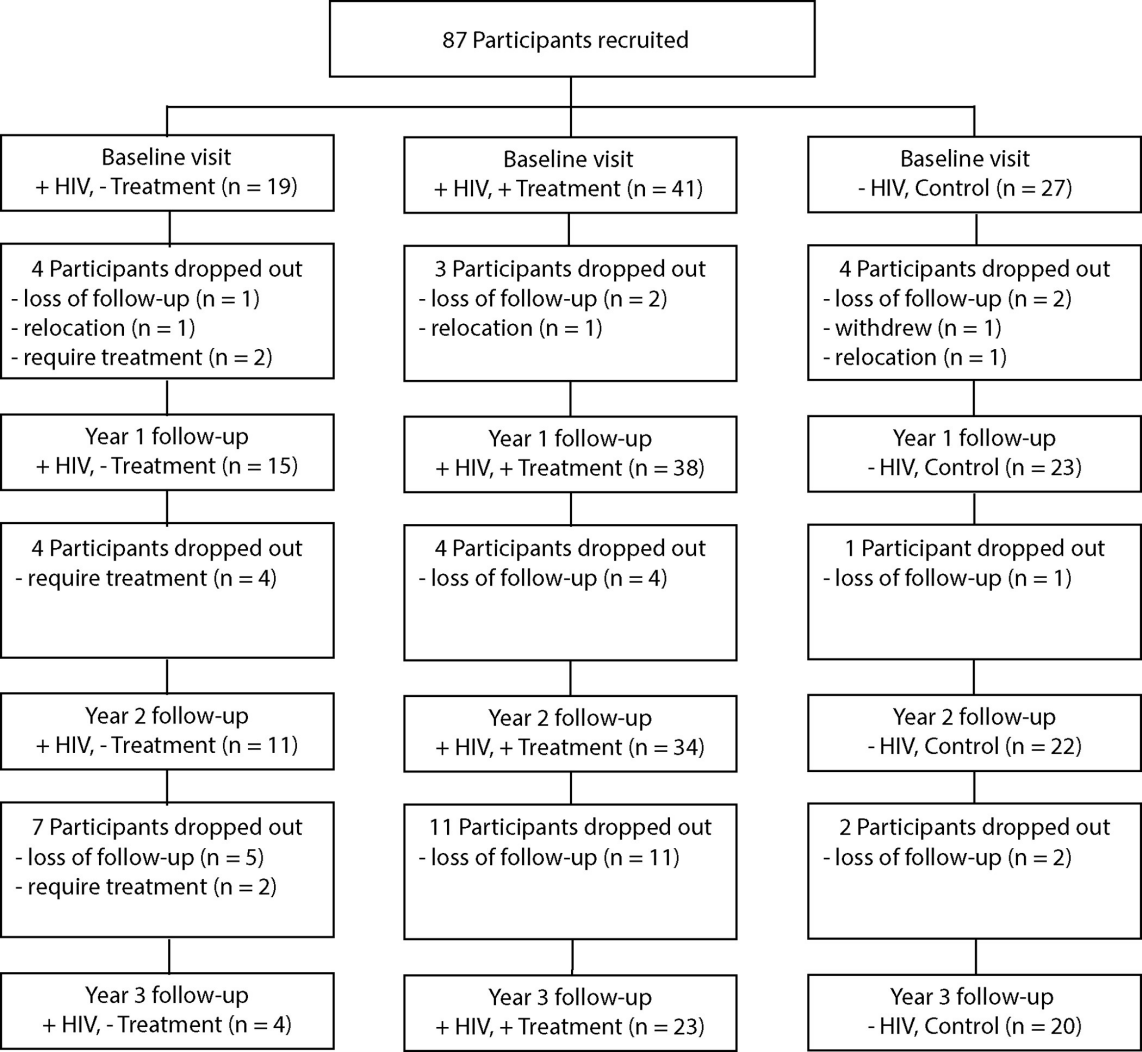
Consort diagram of the study population.

Study participants were recruited from 3 sites, a) Infectious Diseases Outpatient Department (including referral of research participants from General Practice clinics with a high case load of people diagnosed with HIV infection) at The Alfred Hospital, Melbourne, Victoria, Australia; b) The George Washington University HIV Clinic, Washington D.C, USA; or c) Bellvitge University Hospital, Barcelona, Spain. Subjects included in HIV-negative group were recruited from healthy volunteers at sites a) and b). Exclusion criteria were age (under 18 or over 60 years), current lipid-lowering medication (including fish oils), history of Familial Hypercholesterolemia or other form of familial dyslipidemia, HCV infection, impaired liver function, alcohol consumption >250 g (>30 units) per week, and confirmed diagnosis of coronary artery disease, carotid/cerebral artery disease or peripheral artery disease. All participants provided written informed consent and the study was approved by the Alfred Hospital Human Research and Ethics Committee (#377/10), George Washington University IRB (#061030) or Hospital Universitario de Bellvitge IRB (#IRB00005523), respectively. The study complied with principles of Declaration of Helsinki. The flow chart of patient recruitment is presented in Fig 1.

At baseline, participants were asked to complete a questionnaire on the presence or absence of lipodystrophy (self-assessed), smoking history, any alcohol consumption, level of physical activity, current use of recreational drugs and medical history. Prior to the baseline visit, HIV-positive participants had made decisions with their treating clinician with regard to commencing treatment or not, based on their viral load, CD4+ T cell counts and percentage. They were allocated into treated and untreated groups, and recruitment continued with the aim of recruiting 30 participants on each group. The decision of whether each individual participant in HIV-positive untreated group remains untreated or commences treatment was made by the treating physician according to the national guidelines at the time of the decision.

Participants were followed-up annually for 3 years, at each visit plasma samples, IMT measurements, anthropometric measurements (height and weight), blood pressure and pulse rate were collected. Twenty mLs of blood was collected into EDTA tubes after a 10 h fast, and plasma was obtained by low speed centrifugation, aliquoted and frozen at −80°C.

### Intima-Media thickness

At every visit, participants’ carotid arteries were assessed for carotid wall intima-media thickness (cIMT) by ultrasound using Philips iE33 with 11 MHz Linear transducer as previously described [24]. cIMT was defined as the distance between lumen-intima interface and media-adventitia interface and was measured from the 2D high resolution digital images obtained using an automated border-detection algorithm (Philips QLAB, Netherlands). cIMT was determined in areas identified as without plaque by 6 duplicate consecutive measurements made 1–2 cm proximal to the carotid bulb. IMT of both right and left carotid arteries were measured and mean value of right and left carotid artery IMT was used. All measurements were performed by the same site operator blinded to the subjects’ identity at all time points. Coefficients of variation for repeated measures was 5.7%.

### Plasma metabolic profile

Measurements of plasma levels of total cholesterol (TC), high density lipoprotein cholesterol (HDL-C), low density lipoprotein cholesterol (LDL-C), apolipoproteinA-I (apoA-I), apolipoprotein-B (apoB) and triglycerides (TG) were done using the COBAS Integra 400 Plus blood analyser (Roche Diagnostics, Switzerland). Plasma levels of glucose were determined by a Colorimetric assay kit (Cayman Chemical, USA) using 1/5 dilutions, and plasma levels of insulin were determined by ELISA (ALPCO, USA). Plasma levels of LCAT were determined by ELISA (Cell-Biolabs Inc., USA) using 1/10 dilutions and those of CETP were determined by ELISA (Cell-Biolabs Inc., USA) using 1/8 dilutions. hsCRP was determined by ELISA (R&D Systems, USA) using 1/2,500 dilutions; Lp(a) in plasma was determined by ELISA (Mercodia, Sweden) using 1/101 to 1/404 dilutions. Homeostatic model assessments for insulin resistance (HOMA-IR) and insulin sensitivity (HOMA-IS) [25] were calculated for each participant.

Viral load, CD4+ T cell counts, CD4 percentage, and HbA1c were measured using commercial kits and were obtained from participants’ medical records provided to us by the individual recruitment sites.

### HDL sub-populations

The distribution of apoA-I among HDL sub-populations was analysed by non-denaturing PAGE (Thermo Fisher Scientific, USA) and immunoblotting using polyclonal anti-human apoA-I as previously described [26].

### Cholesterol efflux

Cholesterol efflux assay was performed as previously described [27]. Briefly, THP-1 monocytes were differentiated with 100 ng/ml phorbol 12-myristate 13-acetate (PMA; Merck) and incubated in serum-containing RPMI-1640 (Thermo Fisher Scientific, USA) with 0.6 μCi/ml [^3^H]-cholesterol (American Radiolabeled Chemicals Inc., USA) for 72 h. Cells were then incubated for 18 h in serum-free RPMI-1640 containing 4 μM LXR agonist TO-901317 (Cayman Chemical, USA). Cholesterol efflux was performed using 1.1% apoB-depleted plasma for 2 h. ApoB-depleted plasma was obtained after precipitation of apoB-containing lipoproteins with Dextran Sulfate (Merck, USA) as previously described [26].

### RNA isolation and small RNA deep sequencing

RNA from plasma (400 μl) was extracted using the Qiagen miRNeasy mini kit with the use of TRIzol-LS (ThermoFisher) for organic phase separation and was analysed by an Agilent 2100 Bioanalyzer using Small RNA Chips (Agilent Technologies). Small RNA libraries were constructed from total isolated RNA using the Ion Total RNA-Seq Kit V2 (Life Technologies, Australia) and ligated to adapters containing a unique index barcode according to the manufacturers’ protocol. The yield and size distribution of the small RNA libraries were assessed using the Agilent 2100 Bioanalyzer instrument with the DNA 1000 chip (Agilent Technologies). Equally pooled libraries were prepared for deep sequencing using the Ion Chef system (Life Technologies) and sequenced on the Ion Torrent Ion S5 using Ion 540 chips (Life Technologies). Pre-processing of reads, removal of adapters and barcodes were performed using the Torrent Suite (v.5.8.0). Sequences were analyzed for quality control (FASTQC), aligned to the Human genome (HG19) using the Torrent Suite, and files transferred to Partek Genomic Suite and Flow (Partek Incorporated, Singapore) for mapping against miRBase V.20 and normalization to reads per million reads (RPM). miRNAs identified with at least 30 RPM were used for further analysis on Partek Genomic suite which included statistical analysis and hierarchical clustering.

### Statistical analysis

Descriptive statistics (mean ± SD) were calculated for all the variables of interest at baseline. Longitudinal differences between and within groups for primary and secondary outcomes were assessed using mixed-models for repeated measures (MMRM). The MMRM model included group, time point, group by time point interaction, age, sex, BMI, as well as adjustment for baseline measurements by including baseline as a covariate. The model used an autoregressive variance-covariance structure to model within-individual errors. This model was used to analyse all outcomes. Correlations between outcomes were assessed by Pearson Product Movement correlation. Bonferroni correction was performed to adjust for multiple comparisons. Results were reported as mean ± SEM unless indicated otherwise. Statistical analyses were performed using Stata 13 (StataCorp LLC, USA). Sample size calculations for this study were based on previous evidence for primary outcome measures (expected minimum difference = 10%, expected coefficient of variation = 20%, α = 0.05, power > 0.8 for 3 groups) [21].

## Results

### Participants

Three groups of participants were recruited and investigated in this study. At the time of recruitment, the first group consisted of 27 HIV-negative subjects (control group). The second group consisted of 19 HIV-positive treatment-naïve participants who remained untreated for the duration of the study (36 months) (untreated group), while the third group consisted of 41 HIV-positive treatment-naïve patients who initiated treatment with an NNRTI-based regimen at the beginning of the study and continued treatment for the duration of the study (treated group). There were losses in follow up in all three groups during the course of the study (Fig 1). The number of participants in the HIV-negative group reduced to 23 after 12 months, 22 after 24 months, and 20 after 36 months. The number of participants in the HIV-positive treated group reduced to 38 after 12 months, 34 after 24 months, and 23 after 36 months. The number of participants in the HIV-positive untreated group reduced to 15 after 12 months, 11 after 24 months, and 4 after 36 months; due to limited number of participants, only analysis of HDL metabolism is available for the 36 month time-point in this group. The main reason for the high rate of drop out in this group was initiation of ART as medically indicated.

Baseline characteristics of the participants are shown in Table 1. All but 2 participants (at the Alfred Hospital site) were males and there was no statistically significant difference between the groups in BMI, systolic and diastolic blood pressure and pulse rate. There were higher rates of smoking and use of recreational drugs in both HIV-positive groups. HIV-positive subjects had a lower prevalence of alcohol consumption; most of the participants did not report lipodystrophy. HIV-positive treated participants were marginally younger than HIV-negative participants. All outcomes were therefore adjusted for age during the analysis.

**Table 1:** Baseline anthropometric and disease related variable.

Variable	HIV-negative	HIV-positive untreated	HIV-positive treated
Number	27	19	41
Age (y)	42 ± 10	39 ± 10	37 ± 9*
Gender (M:F)	27:0	18:1	40:1
BMI (kg/m^2^)	25.9 ± 4.0	25.3 ± 4.1	24.1 ± 3.2
CD4+ T cell count (cells/μl)	N/A	700 ± 284	437 ± 232
CD4%	N/A	30.1 ± 10.2	23.3 ± 10.1
Viral load (copies/ml)	N/A	35,000 ± 53,000	73,000± 109,000
Systolic BP (mm Hg)	117 ± 10	122 ± 16	122 ± 13
Diastolic BP (mm Hg)	73 ± 11	77 ± 11	76 ± 8
Pulse rate (Beats/min)	70 ± 6	73 ± 15	73 ± 14
Current smoking (y:n:u)^1^	1:26:0	10:9:0**	10:30:1*#
Alcohol Consumption (y:n:u)^1^	27:0:0	11:8:0***	33:7:3*
Exercise (y:n:u)^1^	23:4:0	9:5:5	21:12:8
Lipodystrophy (y:n:u)^1^	1:26:0	1:13:5	3:30:8
Recreational Drugs (y:n:u)^1^	2:25:0	9:10:0**	16:24:1**

Unadjusted values, Mean ± SD are shown

^1^y:n:u = yes: no: unknown (no answer).

*p<0.05 *versus* HIV-negative

**p<0.01 *versus* HIV-negative

***p<0.001 *versus* HIV-negative

#p<0.05 *versus* HIV-positive untreated

### Progression of HIV disease

**Fig 2:**
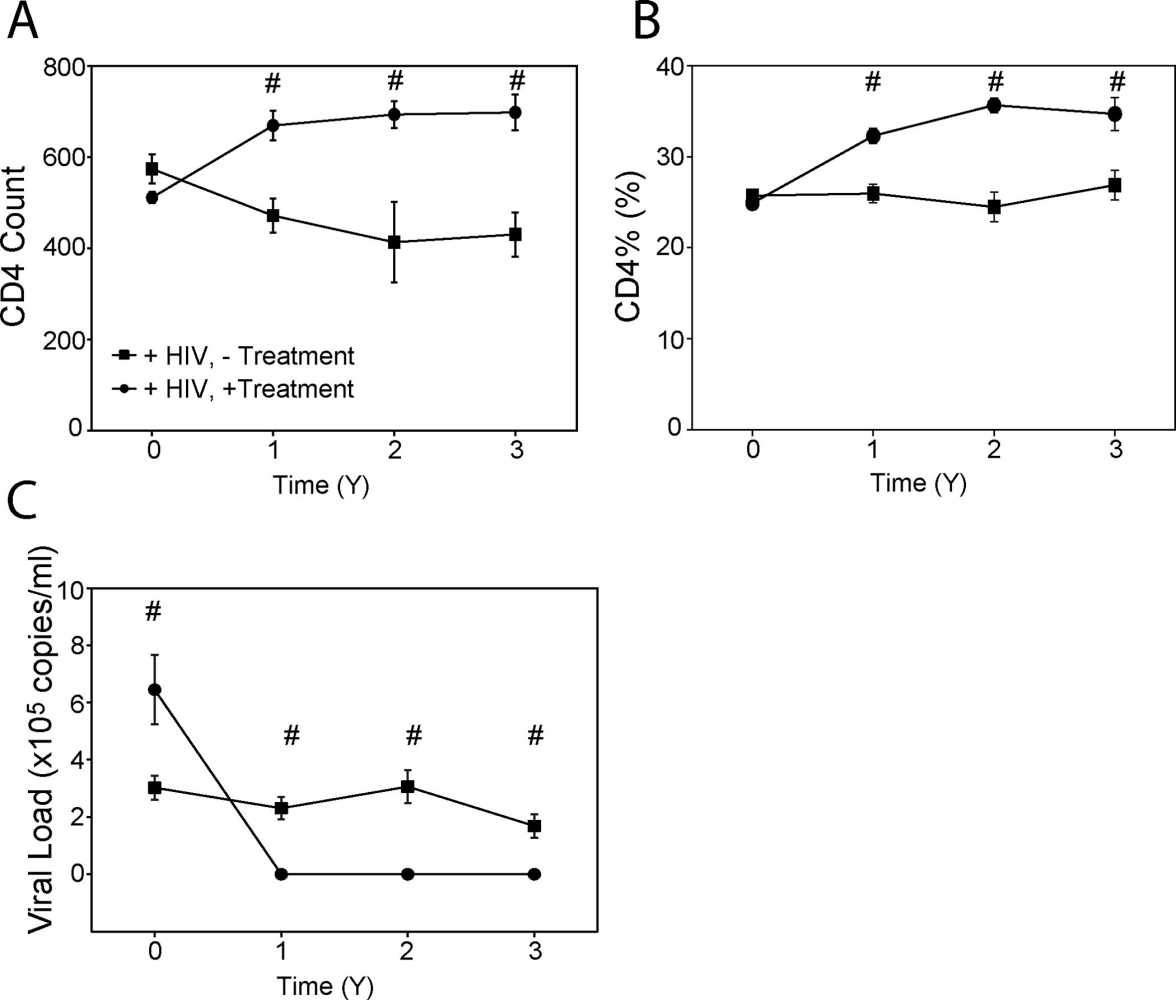
Changes in CD4+ T lymphocytes and HIV viral load between treated and untreated HIV positive participants. **A**–CD4+ cell count; **B**–CD4+ cell percentage; **C**–Plasma viral load. “+HIV,—Treatment”–HIV-positive untreated group; “+HIV, + Treatment”–HIV-positive treated group. ^#^p< 0.05 *versus* HIV-positive untreated group.

At baseline, there was no difference in CD4+ T cell counts or percentages between both HIV-positive groups, however, there was significantly higher viral load in “treated” group (Table 1); the latter was an indication to commence treatment. As expected, after initiation of ART, both CD4+ T cell counts and CD4+ T cell percentage in patients’ blood increased, while viral load decreased to undetectable levels, and these parameters remained stable for the duration of the study (Fig 2A–2C). In the untreated group, CD4+ T cell count gradually declined with time, but CD4+ T cell percentage and viral load remained stable (Fig 2A–2C). There was a statistically significant difference between treated and untreated subjects in all three parameters at all time points after commencement of ART. It is important to recognize a selection bias in assessing progression of HIV disease in untreated subjects, as patients with increasing viral load and declining CD4+ cell counts commenced treatment and were excluded from further follow up.

### Progression of atherosclerosis and diabetes

**Fig 3:**
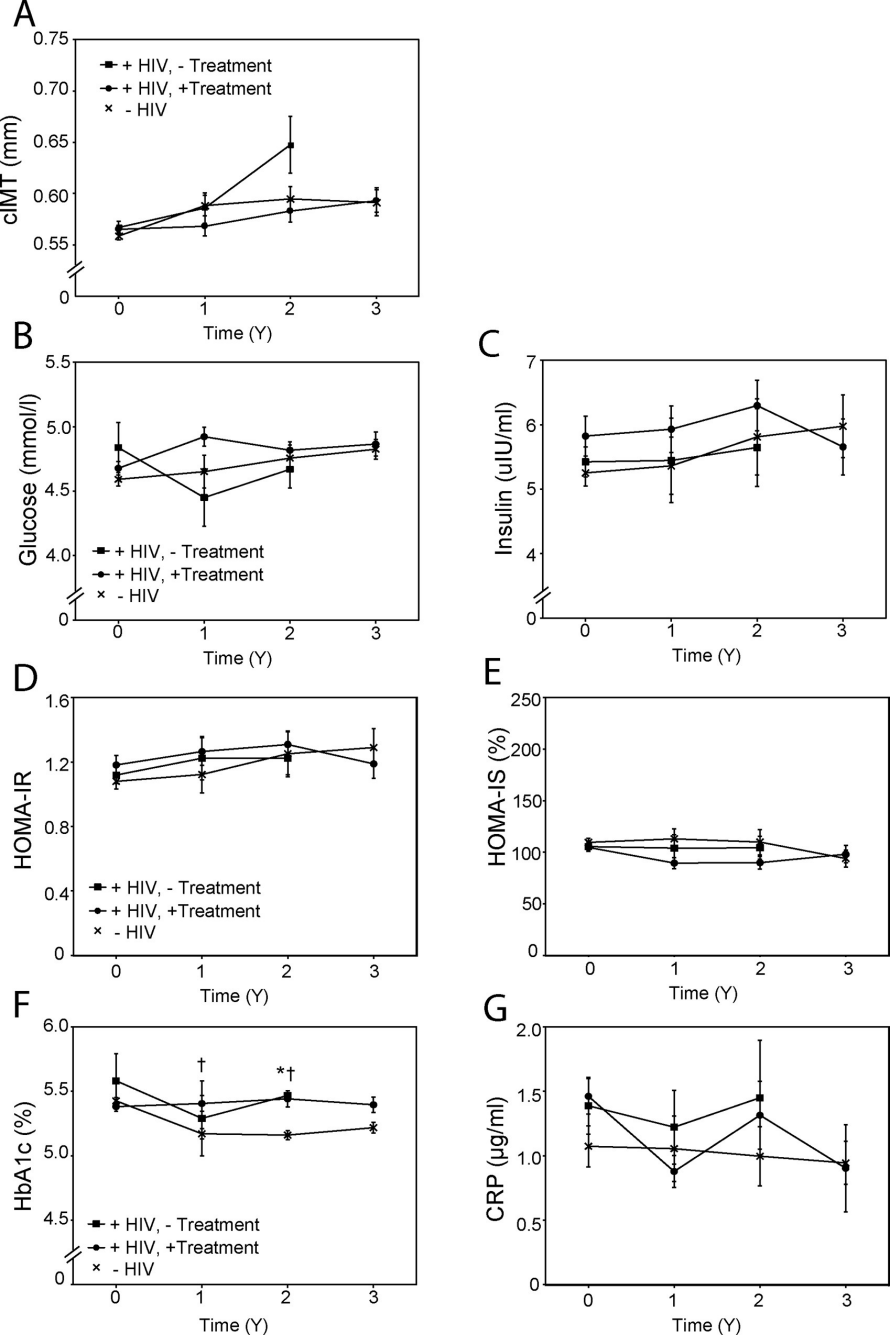
Progression of atherosclerosis and diabetes. **A**–cIMT; **B—**Plasma glucose concentration; **C**–Plasma insulin concentration; **D**–HOMA insulin resistance index; **E–**HOMA insulin sensitivity index; **F**–Plasma HbA1c concentration; **G**–Plasma hsCRP concentration. “+HIV,—Treatment”–HIV-positive untreated group; “+HIV, + Treatment”–HIV-positive treated group; “-HIV”–HIV-negative group. *p<0.05 HIV-negative *versus* HIV positive untreated group; ^†^p<0.05 HIV-negative *versus* HIV-positive treated group.

Progression of atherosclerosis was assessed by monitoring changes in cIMT. There was no statistically significant difference in mean cIMT between the groups at baseline. In HIV-negative subjects cIMT rose slightly after 1 year (p<0.01 *versus* baseline), and no further rise was observed after 2 and 3 years (Fig 3A). In treated HIV-positive patients cIMT rose slightly after 3 years (p<0.05 *versus* baseline), and there was no statistically significant difference *versus* the control (Fig 3A). In untreated HIV-positive patients mean cIMT rose substantially after 2 years (p<0.05 *versus* baseline and 1 year).

Development of diabetes was assessed by measuring fasting levels of glucose, insulin and HbA1c and by calculating HOMA indices. No participant entered the study with a clinical diagnosis of diabetes, and at baseline there was no statistically significant difference between the groups in any of these parameters. Mean glucose and insulin levels remained stable in all three groups for the duration of the study (Fig 3B and 3C). Consequently, no statistically significant changes in HOMA-IR and HOMA-IS were detected (Fig 3D and 3E). HbA1c levels in HIV-negative subjects were reduced during the study, while remaining stable in both HIV-positive groups. Mean HbA1c levels in the HIV-positive groups were higher compared with the HIV-negative group after 2 years of follow up (Fig 3F).

There was considerable variability in mean hsCRP levels with no statistically significant differences found between the groups at the baseline or during follow up (Fig 3G).

### Lipids and lipoproteins

**Fig 4:**
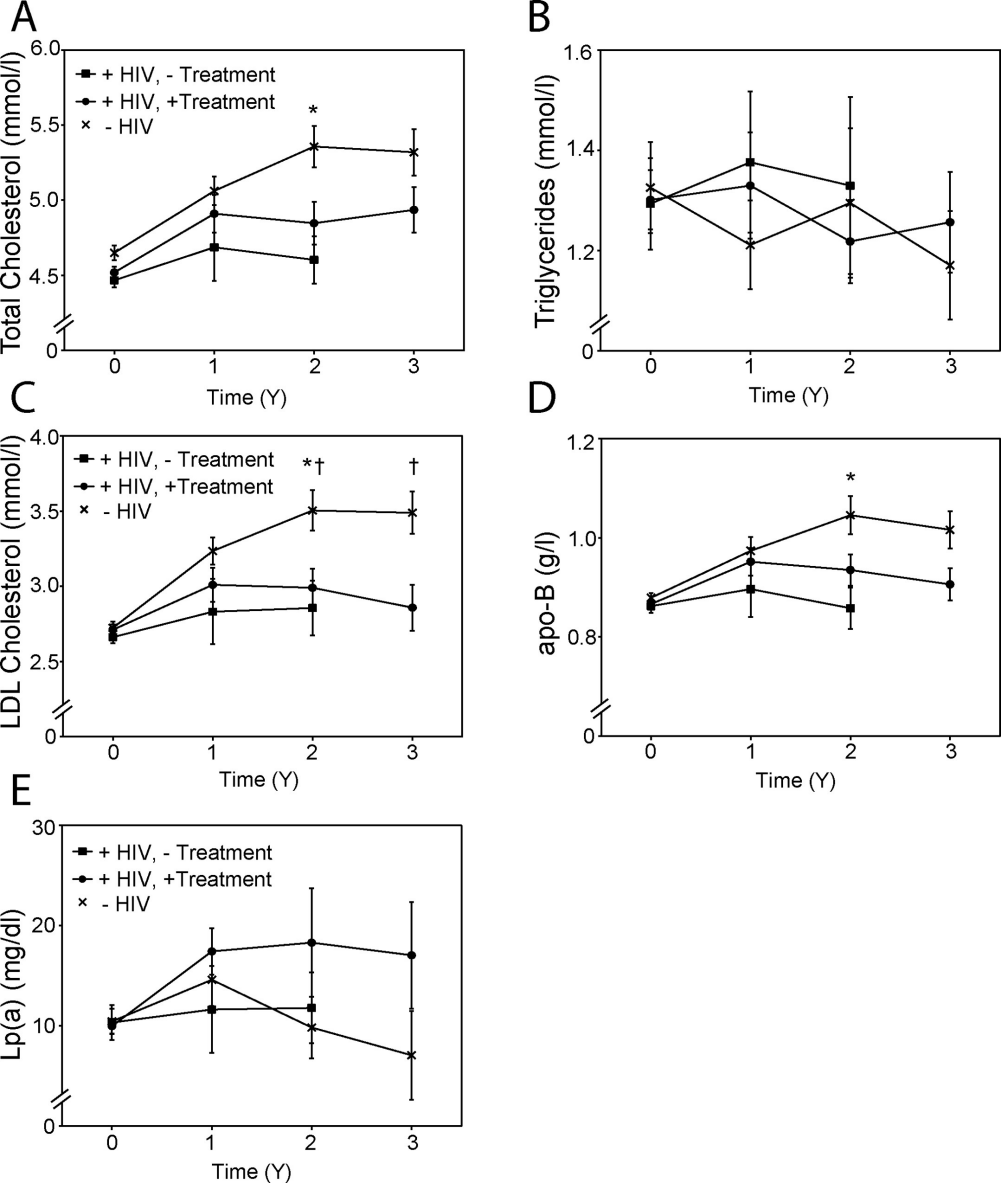
Lipids and lipoproteins. **A**–Plasma total cholesterol levels; **B**–Plasma triglyceride levels; **C**–Plasma LDL cholesterol levels; **D**–Plasma apoB levels; **E**–Plasma Lp(a) levels. “+HIV,—Treatment”–HIV-positive untreated group; “+HIV, + Treatment”–HIV-positive treated group; “-HIV”–HIV-negative group. *p<0.05 HIV-negative *versus* HIV positive-untreated group; ^†^p<0.05 HIV-negative *versus* HIV-positive treated group.

There was no difference in mean plasma levels of total cholesterol between the groups at the baseline (Fig 4A). Over 2 and 3 years of follow up the level of total cholesterol in plasma of HIV-negative subjects increased by 15.2% and 14.4%, respectively (p<0.001). During the same period, plasma total cholesterol level in HIV-positive treated patients increased by 7.3% (p = 0.08) and 9.2% (p<0.05). There was no significant change in plasma total cholesterol level in HIV-positive untreated patients. After 2 years of follow up plasma total cholesterol levels were higher in HIV-negative subjects than in HIV-positive untreated patients (Fig 4A). There was no difference in mean plasma total triglyceride level between the groups at the baseline or during follow up, and total triglyceride content did not significantly change during follow up in all groups (Fig 4B).

Changes in mean plasma low density lipoprotein cholesterol (LDL-C) levels were similar to those of total cholesterol, with no difference between the groups at baseline. A gradual increase in mean plasma LDL-C in the HIV-negative group (28%, p<0.01 at 2 and 3 year time points) was found, a moderate increase in LDL-C levels was observed in the HIV-positive treated group (10%, p<0.05 after 2 years, but no significant increase after 3 years) (Fig 4C). There was no significant change in LDL-C levels in HIV-positive untreated group during follow up. Plasma LDL-C levels were higher in HIV-negative subjects than in both groups of HIV-positive participants after 2 years, and higher than in HIV-positive treated patients after 2 and 3 years (Fig 4C). Changes in mean plasma levels of apoB mirrored those in LDL-C, except that the between group differences after 3 years of follow-up did not reach statistical significance (Fig 4D). There was a trend for mean plasma levels of Lp(a) to be higher in HIV-positive treated patients compared to the other two groups at all time points except at baseline, however, the variability was high and the differences did not reach statistical significance (Fig 4E).

### High density lipoprotein and parameters of reverse cholesterol transport

**Fig 5:**
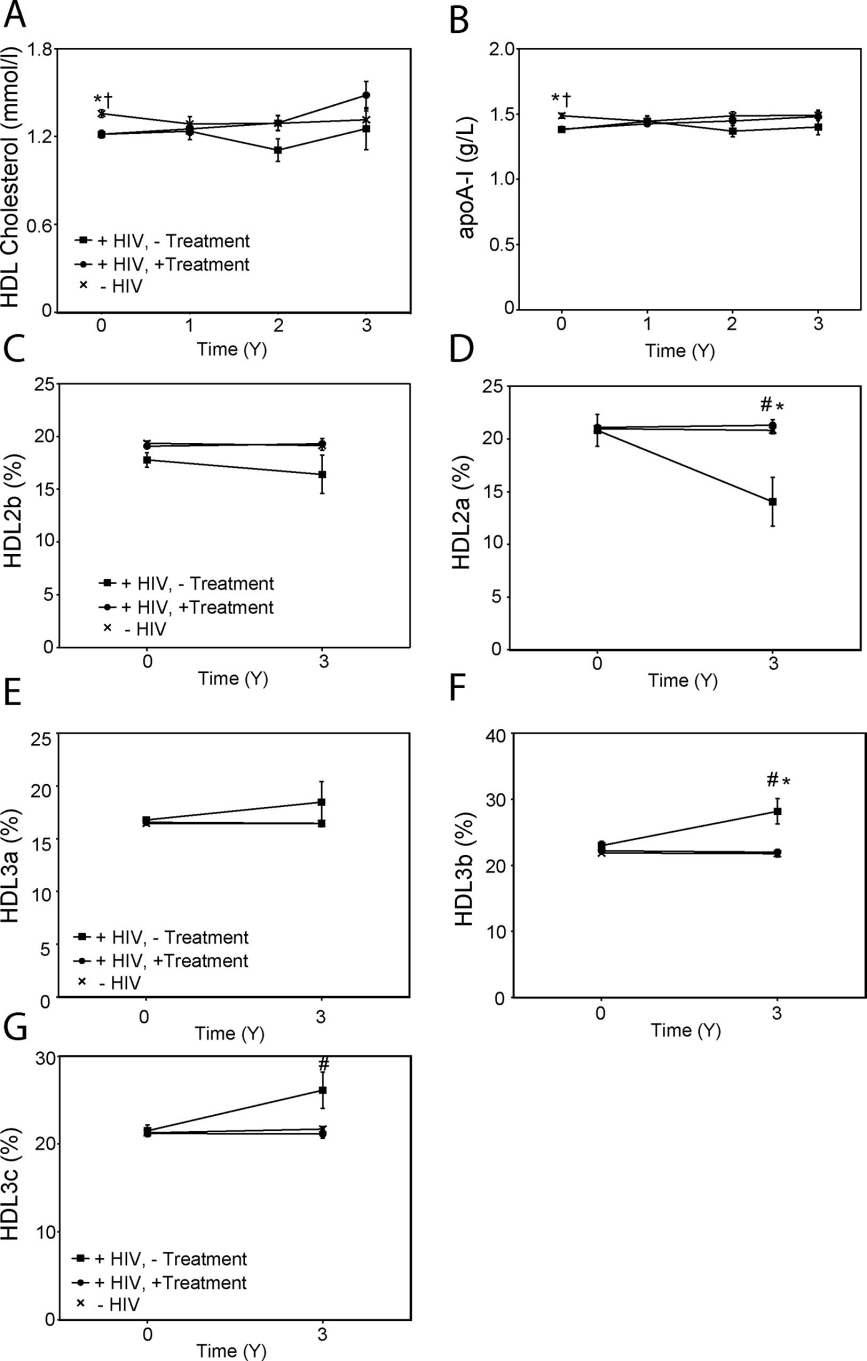
High density lipoproteins. **A**–Plasma HDL cholesterol levels; **B**–Plasma apoA-I levels; **C**—Proportion of HDL2b particles in total HDL; **D**—Proportion of HDL2a particles in total HDL; **E**—Proportion of HDL3a particles in total HDL; **F**—Proportion of HDL3b particles in total HDL; **G**—Proportion of HDL3c particles in total HDL. “+HIV,—Treatment”–HIV-positive untreated group; “+HIV, + Treatment”–HIV-positive treated group; “-HIV”–HIV-negative group. ^#^p< 0.05 HIV-positive treated *versus* HIV-positive untreated group; *p<0.05 HIV-negative *versus* HIV-positive untreated group; ^†^p<0.05 HIV-negative *versus* HIV positive treated group.

At baseline, mean plasma levels of high density lipoprotein cholesterol (HDL-C) and apolipoprotein A-I (apoA-I) in the HIV-positive groups were lower compared to those in HIV-negative subjects (Fig 5A and 5B; p<0.05). There were no statistically significant differences between the groups at all follow-up points (Fig 5A and 5B).

We used non-denaturing gradient gel electrophoresis to analyse changes in the distribution of apoA-I among HDL particles of different sizes between baseline and after 3 years of follow up. We found no difference between the groups at baseline, but a significant increase in proportion of small HDL3b and HDL3c particles at the expense of large HDL2a particles in HIV-positive untreated patients when compared to the other two groups (Figs 5C–5G).

**Fig 6:**
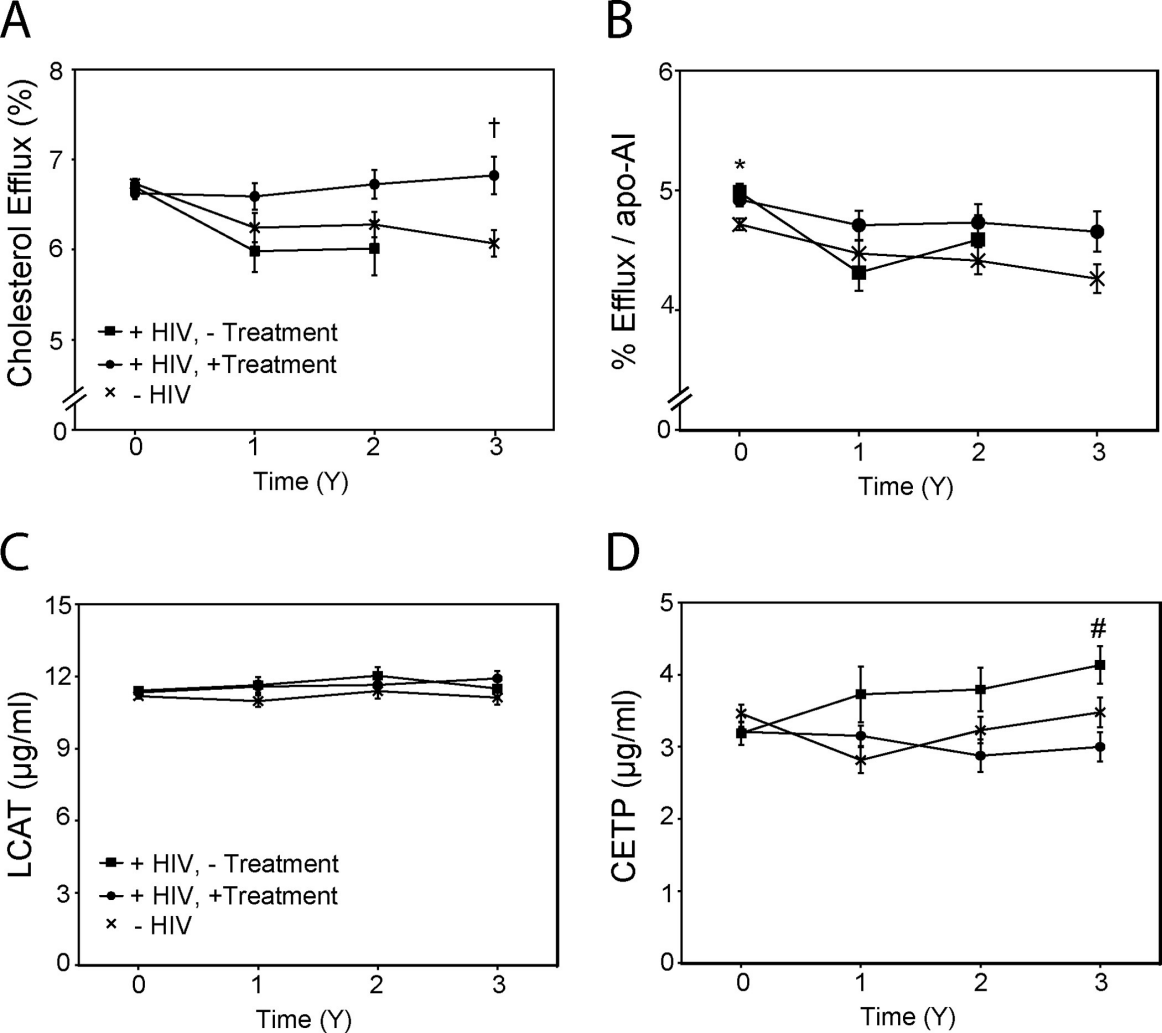
Reverse cholesterol transport. **A**–HDL functionality defined as the capacity of HDL (apoB-depleted plasma) to support cholesterol efflux from THP-1 macrophages; **B–**Functionality of individual HDL particles (defined as capacity of apoB-depleted plasma to support cholesterol efflux normalised to apoA-I level); **C**–Plasma concentration of LCAT; **D**–Plasma concentration of CETP. “+HIV,—Treatment”–HIV-positive untreated group; “+HIV, + Treatment”–HIV-positive treated group; “-HIV”–HIV-negative group. *p<0.05 HIV-negative *versus* HIV-positive untreated group; ^†^p<0.05 HIV-negative *versus* HIV-positive treated group, ^#^p<0.05 HIV-positive treated versus HIV-positive untreated group.

Functionality of HDL was measured as a capacity of 1.1% serum depleted of apoB-containing lipoproteins to support cholesterol efflux from differentiated THP-1 macrophages (see Materials and Methods for details). There was no difference in HDL functionality between the groups at baseline (Fig 6A). HDL functionality remained unchanged in the HIV-positive treated group for the duration of the study, while gradually declined in the HIV-negative group, and in the HIV-positive untreated group (Fig 6A; p<0.05 baseline *versus* 2 and 3 years). There was statistically significant difference between the HIV-positive treated and HIV-negative groups after 3 years of follow up (Fig 6A). We also calculated functionality of individual HDL particles by relating values of cholesterol efflux to concentration of apoA-I (Fig 6B). Similar to the overall HDL functionality, this parameter declined during follow up in HIV-negative group (Fig 6B, p<0.01), but not in treated HIV-positive group. The variability of this parameter was high and the only statistically significant difference between the groups was that between HIV-negative and HIV-positive untreated groups at baseline (Fig 6B).

There was no difference in plasma concentration of lecithin cholesterol acyltransferase (LCAT) between the groups or within the groups during follow up (Fig 6C). Plasma concentration of cholesteryl ester transfer protein (CETP) was similar for all three groups at baseline and did not change during follow up in HIV-negative and treated HIV-positive groups, but gradually increased in the HIV-positive untreated participants (Fig 6D).

### Plasma microRNAs

**Fig 7:**
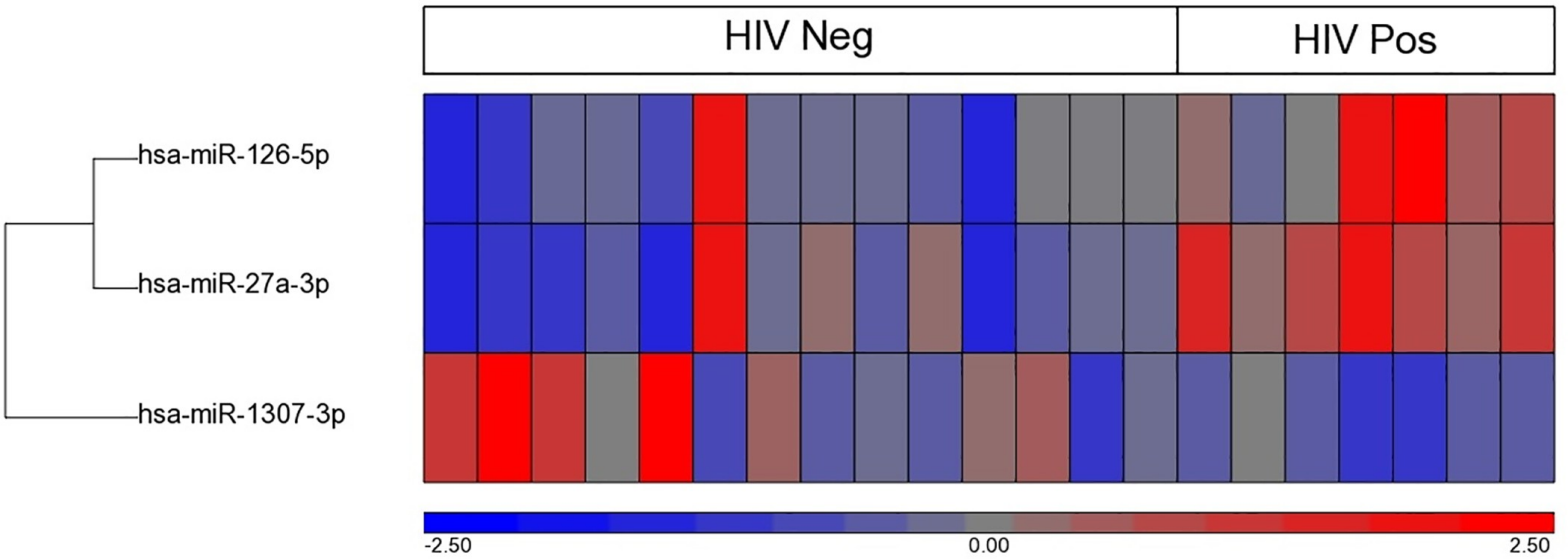
Hierarchical clustering of significantly differentially expressed miRNA in HIV-positive and HIV-negative samples.

We and others have previously suggested that the effects of HIV on lipid metabolism may be mediated in part through differences in microRNAs released into bloodstream [28, 29]. To determine whether the effects of HIV on lipid metabolism are mediated through microRNAs, we analysed differences in plasma microRNA profile in a subgroup of HIV-negative (n = 14) and HIV-positive (n = 7) participants at a single time-point (6 months). Small RNA sequencing revealed 72 highly expressed microRNAs displaying more than 30 reads per million (RPM) across all samples (S1 File). Three microRNAs were shown to be significantly differently expressed between HIV-positive participants compared to HIV-negative group, with differences greater than 1.5 fold and p<0.05 (Fig 7 and Table 2). Two of these microRNAs, hsa-miR-27a-3p and hsa-miR-126-5p, were overrepresented, while hsa-miR-1307-3p was under-represented in plasma of HIV-positive subjects (Fig 7 and Table 2).

**Table 2:** MicroRNAs differentially abundant in plasma of HIV-positive *versus* HIV-negative participants.

microRNA	P value	HIV+/HIV- ratio
hsa-miR-27a-3p	0.00112	1.58
hsa-miR-126-5p	0.01038	1.58
hsa-miR-1307-3p	0.04281	0.55

Raw sequencing reads were aligned to the human genome (HG19) and mapped to miRBase V.20. Reads were then normalised to reads per million (RPM) and miRNA species with greater than 30RPM were used for ANOVA analysis. Significantly expressed miRNA expressing greater than 1.5 fold changes and p-value < 0.05 are shown here. Red indicates high expression and blue indicates low expression.

### Associations

To investigate an association between the severity of HIV infection and changes in surrogate markers of atherosclerosis or metabolic parameters, we analysed correlations between all metabolic parameters and IMT *versus* CD4+ T cell count and HIV viral load. For this analysis we combined all data for both groups of HIV-positive patients. We found a statistically significant negative association between viral load and plasma levels of total cholesterol (r = -0.21, p<0.001), LDL cholesterol (r = -0.13, p<0.001), HDL cholesterol (r = -0.19, p<0.001), apoA-I (r = -0.24, p<0.001) and apoB (r = -0.16, p<0.001). We also found a negative association between viral load and cIMT (r = -0.18, p<0.05), but this was mainly due to a sharply decreased viral load combined with modest rise in IMT in treated HIV-positive patients. We found a statistically significant positive association between CD4+ T cell count and proportion of HDL3c particles and combined HDL3 (HDL3a+HDL3b+HDL3c) (r = 0.28, p<0.01), and a negative association between CD4+ T cell count and proportion of combined HDL2 (HDL2a+HDL2b) particles (r = -0.28, p<0.01).

## Discussion

In this study, we investigated metabolic dysfunction and atherosclerosis in people newly diagnosed with HIV infection who commenced antiretroviral therapy (ART) or remained untreated for 3 years. This study was performed prior to the recommendation that all HIV-positive patients commence ART immediately after HIV diagnosis regardless of CD4 cell count. A significant rise in cIMT was observed in untreated HIV-infected patients after 2 years. In uninfected group, there was a modest rise in cIMT after 1 year, which did not progress further, and in treated patients a modest rise was seen after 3 years. This finding indicates that progression of atherosclerosis, as assessed by changes in cIMT, seen in HIV-positive patients, was mitigated by ART. This is consistent with a suggestion that HIV infection itself, rather than side-effects of ART, is a major cause of elevated cardiovascular risk in HIV-positive patients [10, 30]. It is also consistent with the view that controlling HIV disease with modern ART regimens effectively restrains progression of atherosclerosis without bringing an additional iatrogenic cardiovascular risk. The changes in atherosclerosis progression took at least two years to manifest, consistent with no change in cIMT after one year of follow up reported in our previous study [21]. Differences in lifestyle factors between HIV-negative and treated HIV-positive subjects apparently did not contribute significantly to the progression of atherosclerosis, a finding consistent with our previous report [13]. Recognizing limitations of IMT as a predictor of future cardiovascular events [31], our findings tentatively support a hypothesis that HIV infection contributes to the pathogenesis of atherosclerosis, as proposed in a number of cross-sectional and prospective studies [2, 5, 14–16, 20–22] and confirmed in recently published meta-analysis [9].

Analysis of diabetes markers, plasma glucose, insulin and HbA1c levels as well as HOMA indices, showed no significant increase in progression to diabetes related to HIV infection, ART or lifestyle factors. This is in contrast to several reports showing increased risk of diabetes in HIV-positive patients [32, 33]; the differences likely attributable to exposure to older treatment regimens, age, lifestyle factors or genetic background of the study population. There was also no effect of HIV or ART on a marker of inflammation, hsCRP, although variability of this parameter was high. This finding does not rule out low grade inflammation in HIV-positive patients.

We found a consistent difference in plasma levels of total cholesterol, LDL cholesterol and apoB between HIV-negative and HIV-positive participants. Hypocholesterolemia in untreated HIV-positive patients has been reported previously [34, 35] while HIV-associated hypercholesterolemia was predominantly attributed to the PI-containing treatment regimens [35, 36], the latter were not used in this study. It appears that the effect on plasma level of LDL was related to HIV and was not alleviated by ART. We did not find any effect of HIV or ART on plasma triglycerides, a finding contrasting with some reports [34, 37] but consistent with previous findings from our laboratory [21] and also consistent with lack of diabetes in our study population. Of note, patients on lipid-lowering or anti-diabetic medications were excluded from the study ruling out the effect of these treatments.

Consistent with findings from our and other laboratories [21, 34, 35, 37, 38], HIV-positive patients had hypoalphalipoproteinemia at baseline. However, with slight decrease of HDL levels in the HIV-negative group and slight increase in HIV-positive groups, the difference disappeared during follow up. Instead, we found redistribution in HDL sub-populations, with increased proportion of smaller HDL particles at the expense of larger HDL particles in untreated HIV-positive patients. This is consistent with our previous findings in SIV-infected monkeys [39] and may be a result of increased plasma levels of CETP in this group, the latter being consistent with our published reports [21, 38]. The changes in HDL functionality were not evident at baseline, consistent with our previous study [21], but progressed during 3 years of follow-up. When normalized to the number of HDL particles, the functionality of HDL gradually declined in all groups and the differences were no longer statistically significant, indicating that changes in overall HDL functionality is a combination of changes in functionality of the particles and in their numbers.

Relative to control group, the HIV-infected groups had higher prevalence of smoking, recreational drug use and lower prevalence of alcohol consumption, lifestyle factors that can affect many parameters measured in this study. However, the two HIV-positive groups were similar for these lifestyle factors and most differences seen in HIV-infected untreated group were not seen in HIV-infected treated group over the course of the study suggesting that the differences between the two HIV-infected groups were unlikely to be due to these lifestyle factors.

An interesting finding of this study is the effect of HIV infection on plasma microRNA profile. Three most strongly affected microRNAs were found to regulate pathways related to the development of atherosclerosis and metabolic dysfunction. MiR-27 plays a prominent role in lipid metabolism: it is one of the key regulators of the expression of ABCA1 and LDL receptor [40, 41] and is also involved in regulation of adipogenesis [42]. Polymorphism in miR-27 was found to be associated with higher body mass in preeclamptic HIV-infected women [43] MiR-126 is an important element of anti-inflammatory and anti-atherogenic response to the lipid-induced inflammation [44, 45], but was shown to be a positive regulator of inflammatory response in HIV-positive monocytes and was upregulated in chronic HIV infection [46]. Differential expression of miR-1307 in monocytes was identified as a signature of diabetes [47]. Many miRNAs are transported in plasma by HDL, and it is unclear if changes in the abundance of miRNAs are a direct result of infection or of changes in HDL structure induced by infection.

This study has several important limitations. First, the group size was limited, especially that of HIV-positive untreated group. Evolving guidelines demanded initiation of treatment at earlier stages of the disease, depleting this group as the study progressed and introducing a bias by removing patients with more rapid progression of the disease from the untreated group. Thus, negative findings of this study should be treated with caution. Second, we did not differentiate between different treatment regimens. Treatment regimens were determined by the treating physicians and very few patients in HIV-positive treated group received identical regimens; moreover, regimens were frequently changed during the follow up period. Finally, it is important to recognize that cardiometabolic co-morbidities may take more than 3 years to develop and their development may depend on the length of the period between the actual infection, diagnosis and commencement of ART.

Overall, this study demonstrates that HIV infection is associated with disturbances of several aspects of cholesterol metabolism and with advancement in the progression of atherosclerosis. Both of these effects are most likely caused by the virus itself. Modern anti-retroviral therapy had mitigated most of the cardiometabolic effects of HIV, at least in short-term, and had no adverse contribution to the cardiometabolic risk. Lifestyle factors also did not have a significant contribution to the cardiometabolic risk.

## Supporting information

S1 File Database 1. Sequencing database of miRNA in plasma of study subjects.
(XLSX)

S2 File Database 2. Manuscript data.
(XLSX)
